# Ankle joint position sense acuity differences among stroke survivors at three walking ability levels: a cross-sectional study

**DOI:** 10.3389/fneur.2024.1407297

**Published:** 2025-01-06

**Authors:** Jinyao Xu, Jeremy Witchalls, Elisabeth Preston, Li Pan, Gengyuan Zhang, Gordon Waddington, Roger David Adams, Jia Han

**Affiliations:** ^1^Research Institute for Sport and Exercise, University of Canberra, Canberra, ACT, Australia; ^2^Faculty of Health, University of Canberra, Canberra, ACT, Australia; ^3^Department of Rehabilitation, Huashan Hospital, Fudan University, Shanghai, China; ^4^Department of Rehabilitation, Shanghai Xinqidian Rehabilitation Hospital, Shanghai, China; ^5^College of Rehabilitation Sciences, Shanghai University of Medicine and Health Sciences, Shanghai, China

**Keywords:** lower limb, ankle, proprioception, walking ability, unilateral stroke

## Abstract

**Background:**

Despite the importance of lower limb sensation in walking highlighted in systematic reviews, there is limited research investigating the effect of proprioceptive deficits after stroke and any relationship with walking ability.

**Objectives:**

With stroke survivors of different walking ability, this study aimed to (1) explore side (affected/unaffected) and movement direction (inversion/plantar flexion) effects in ankle joint position sense (JPS) acuity, and (2) compare ankle JPS acuity between groups of stroke survivors with different walking ability.

**Methods:**

Seventy subacute stroke survivors were recruited and divided into three groups based on walking ability, as determined by their gait speed on the 10-Meter Walking Test: household (<0.4 m/s), limited community (0.4–0.8 m/s) and community (>0.8 m/s). Ankle JPS acuity was measured by the active movement extent discrimination apparatus (AMEDA).

**Results:**

A significant difference was found between sides, with the AMEDA scores for the unaffected side significantly higher than for the affected side (F_1.67_ = 22.508, *p* < 0.001). The mean AMEDA scores for plantar flexion were significantly higher than for inversion (F_1.67_ = 21.366, *p* < 0.001). There was a significant linear increase in ankle JPS acuity with increasing walking ability among stroke survivors (F_1.67_ = 17.802, *p* < 0.001).

**Conclusion:**

After stroke, ankle JPS acuity on the affected side was lower than the unaffected side. Stroke survivors had higher ankle JPS acuity in plantar-flexion movements, compared with inversion movements. Overall, stroke survivors with higher ankle JPS acuity tended to have higher walking ability, highlighting the importance of ankle JPS acuity in walking ability after stroke. These findings provide new insights into proprioceptive deficits after stroke and their relevance in neurorehabilitation.

## Introduction

Stroke is a leading cause of mortality and results in long-term disability worldwide (1). For stroke survivors, loss of independent walking, especially in community settings, is one of the most disabling aspects of the condition in relation to daily living (2). Only 30–65% of individuals with stroke recover sufficiently to achieve safe and independent walking in the community (2, 3). The recovery of independent walking is challenging because it requires appropriate integration of sensory inputs and adaptive motor output.

One in two individuals after stroke experience sensory deficits (4), with the lower limbs being affected in approximately 50% (5, 6). Difficulty sensing affected lower limbs while walking, particularly the foot and ankle (7) is typically reported after stroke as well as difficulty in detecting the loading force on the affected lower limb (8). Hence, stroke survivors put less loading force on the affected lower limb in standing (9) and use the unaffected side more, for reasons of safety and speed during walking (10). Stroke survivors with sensory deficits also experience reduced coordination in lower limbs (11), which leads to decreased walking speed (12). Further, in individuals with mild to moderate stroke, ankle sensation has been reported as the third greatest contributor, following strength and spasticity, to walking speed (13). Despite the important effect of lower limb sensation on walking that has been highlighted in two systematic reviews, there is limited research investigating the presence of sensory deficits after stroke, and their relationship with walking (14, 15). Therefore, it is important to improve understanding of post-stroke sensory deficits in the lower limbs and thus support mechanisms for addressing the impact of sensory deficits on walking in clinical interventions.

Proprioception is a closed-loop system that involves sensory input, central processing and motor output (16). Specifically, it integrates sensory signals from mechanoreceptors in the muscles, tendons, joints and skin, continuously providing the central nervous system (CNS) with updated information about joint movement and joint position sense (JPS). This feedback allows the CNS to maintain posture and control voluntary movement (17, 18). Loss of proprioception is a common clinical issue after stroke. Previous studies have found that stroke survivors who have proprioceptive deficits in the foot and ankle may demonstrate significant impairments in motor function with the affected lower limb, even if muscle strength remains unaffected (19). Ankle-foot complex proprioception, in particular, has been regarded as an essential sensory component that is critical for the adjustment of ankle position and for coordination of upper body movements when stroke survivors perform balance-related tasks (20). It is therefore plausible that lower limb proprioceptive deficits are associated with reduced walking ability. However, there is little existing literature that examines the impact of proprioceptive deficits of the foot and ankle on walking ability after stroke.

Better understanding of the extent to which proprioceptive deficits and walking ability are associated can help to guide the content of post-stroke rehabilitation. If better ankle JPS acuity is associated with improvement in walking ability, clinical interventions might focus more on sensory training. Thus, the aims of the present study were (1) to explore side (affected/unaffected) and movement direction (inversion/plantar flexion) effects on ankle JPS acuity after stroke and (2) to compare ankle JPS acuity between groups of stroke survivors with different walking ability (household, limited community and community).

## Materials and methods

### Study design

This cross-sectional study was conducted from June 2021 to February 2022 in the Neurology and Rehabilitation Wards of a hospital In Shanghai, China. Using a non-probability convenience sampling method, all eligible stroke survivors admitted during the study period were recruited. Ankle JPS acuity and walking ability were measured during one session in a hospital rehabilitation gym. The study was approved by the Hospital Human Research Ethics Committee (2021-7th-HIRB-025) and written informed consent was obtained from all participants prior to measurement.

All participants received at least an hour of individual exercise, 5 days per week while in hospital. Continued rehabilitation (in-patient and/or out-patient) was provided based on the individual’s goals according to the three-stage rehabilitation network guidelines provided in China (21). In these guidelines, stage 1 is defined as early rehabilitation (within 1 month after stroke onset) in the Stroke Unit, stage 2 is rehabilitation in the recovery period (1 month to 6 months after stroke onset) in a Specialized Rehabilitation Hospital, and stage 3 is chronic rehabilitation (more than 6 months after stroke onset) in the Community Hospital or Home.

### Participants

The sample size was estimated using G*Power 3.1 for a one-way analysis of variance (ANOVA). Based on a previous observational study that used the AMEDA to assess bilateral ankle JPS acuity in subacute stroke survivors (22), this study was powered to detect a difference in ankle JPS acuity of *f* = 0.5, with a significance level of *α* = 0.05. The power was set at 0.9 and the number of groups was set n = 3. This provided a minimum sample size of 54 participants, including at least 18 stroke survivors in each walking ability group.

Participants were enrolled in this study if they (1) had a first occurrence unilateral stroke, (2) had sufficient cognition to follow the instructions required to complete the assessment (defined as obtaining a score greater than 24 on the mini mental state examination - China), (3) were able to walk at least 10 m, and (4) were able to bear weight and generate ankle plantar flexion and inversion movements with or without the support of a handrail. Potential participants were excluded if they had (1) a previous stroke, (2) significant dysphasia, (3) severe unilateral spatial neglect, and (4) any other neurological condition which could affect balance or walking.

### Measurements

After informed consent, individuals with stroke participated in a one-hour assessment session. Both outcome measurements were conducted by two registered neurological physiotherapists, in accordance with standard administration practice as described in previous studies (22, 23).

The walking ability of participants relative to real life activity was determined by gait speed in the 10-Meter Walk Test (10MWT). To measure gait speed during walking, the 10MWT, a functional assessment tool commonly used in clinical practice, was performed and recorded in m/s (23).

The Active Movement Extent Discrimination Apparatus (AMEDA) was used to assess ankle joint position sense acuity. It has shown good to excellent reliability for testing in older adults (24) and has also been validated in stroke survivors (22).

### Protocol

Each participant first underwent the 10MWT and the test was performed without the use of any walking aid. The 14-meter walkway was marked at 0 m, 2 m, 12 m, and 14 m points. Participants were guided to walk at a comfortable, self-selected pace along the walkway. Considering the need for acceleration and deceleration, only the time taken to complete the middle 10 meters (between the 2 m and 12 m points) was recorded. Each trial was timed using a digital stopwatch, with three trials performed in total. The mean gait speed across the three trials was then calculated. Participants were given a short break after each trial. Based on the gait speed results from the 10MWT, participants were categorized into three groups: household (<0.4 m/s), limited community (0.4–0.8 m/s) and community (>0.8 m/s) ambulators. These performance criterion needed for community-level walking ability have been established in previous research (25).

**Figure 1:**
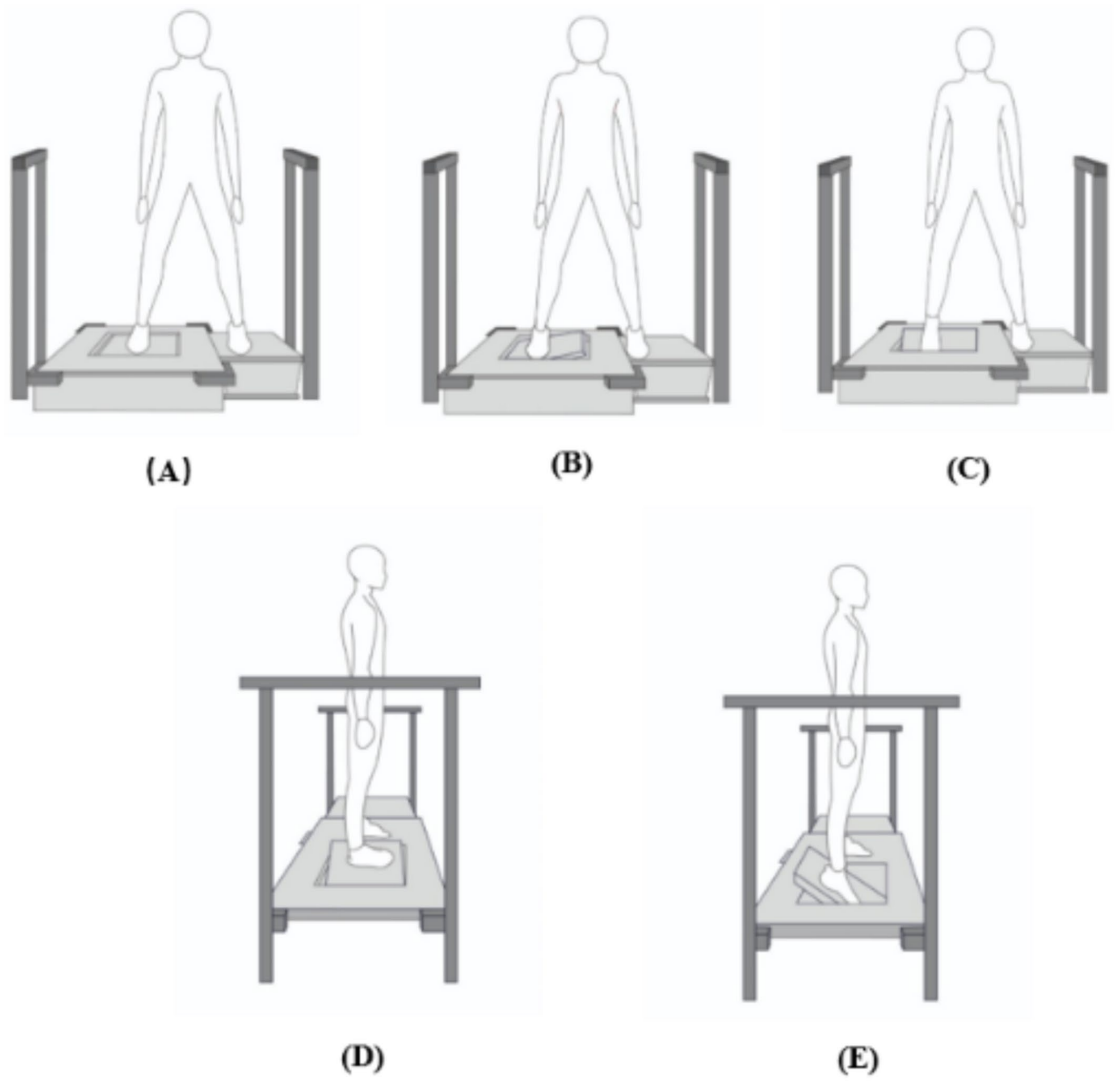
Front and side view of the Active Movement Extent Discrimination Apparatus (AMEDA) set-up. **(A)** Neutral position. **(B)** Front view of inversion movement. **(C)** Front view of plantar flexion movement. **(D)** Side view of inversion movement. **(E)** Side view of plantar flexion movement. After each movement, the participant was required to return the plate to the neutral position and then judge the movement extent.

After 10MWT, the AMEDA was used to test ankle JPS acuity in a weight-bearing, free standing position. Both lower limbs were tested separately for the inversion and plantar flexion movement directions. During the test, participants were required to stand with one foot on a fixed platform, and the other on a moveable platform that could be adjusted so that it was capable of tilting into either inversion or plantar flexion movement directions. Participants were required to stand barefoot and look straight ahead at a point on the wall in front of them, to ensure that judgment was based solely on proprioceptive information. They performed the movement test by actively controlling the tipping of the movable platform to the predetermined stop point (Figure 1).

A mechanism located underneath the platform can generate 4 different levels (10°, 12°,14° and 16°), by changing the depth to which the platform can tilt. Parallel bars were fixed on the floor at both sides of the platform for participants to hold on to for support if they required it during the assessment.

Participants were given a familiarization session before data collection. They performed the movement to each of the 4 different levels, in order, from the shallowest to the deepest. The familiarization session consisted of practice with feeling the depth of movement at each level sequentially, 5 times, for 20 movements in total, to allow participants to appreciate the difference between the 4 levels. Participants thereafter undertook the test by determining which level was set for each of 40 trials that were given in a random sequence, with each of the 4 levels presented 10 times. Participants were required to tilt the platform down, return it to the start position, and immediately report the level to which they sensed the platform to have tilted. No feedback was given throughout the test as to the accuracy of their estimates.

Area under the Receiver Operating Characteristic (ROC) curve (AUC) values were used to represent each participant’s performance in the AMEDA test, reflecting their ability to discriminate small differences in the extent of active ankle plantar flexion and inversion movements. Raw data were entered into a 4 × 4 matrix representing the frequency with which each response was made for each stimulus. Non-parametric signal detection analysis was used to produce pair-wise receiver operating characteristic (ROC) curves, that compared responses between the successive levels 1–2, 2–3, and 3–4 (26). Then, the mean area under the ROC curve (AUC) was calculated using SPSS software v.26, providing each participant with an ankle movement discrimination score. AUC values can range from 0.5 to 1.0, where 0.5 is equivalent to chance responding and 1.0 represents perfect discrimination (22).

### Data analysis

Analyses were performed using SPSS Statistics v.26 (IBM Corporation, Somers, NY), with 0.05 chosen for statistical significance and all figures were generated by GraphPad 8.3.0 (San Diego, CA). The normality of data distributions was assessed using the Shapiro–Wilk test. Normally distributed continuous variables were presented as mean values with standard deviation (SD), while non-normally distributed continuous variables were presented as median and range. The results of these preliminary analyses indicated that the data met the assumptions for parametric testing, supporting the use of ANOVA and *t*-tests.

One-way ANOVA was conducted to compare the demographic characteristics and ankle AMEDA scores among the three walking ability groups. Post-hoc analysis was performed to further examine differences in ankle AMEDA scores between groups.

A 2x2x3 repeated measures analysis of variance (ANOVA) with factors Side (affected and unaffected), Movement (plantar flexion and inversion directions) and Walking Ability (Household, Limited Community and Community) was used to explore the effects of Side, Movement and Walking Ability on ankle JPS acuity after stroke. Post-hoc paired t-test analyses were examined if the effect of Side, Movement, Walking Ability, or their interactions reached significance (*p* < 0.05). Polynomial trend contrast analysis was conducted to examine for significant linear and quadratic trend components in ankle AMEDA scores over the different walking ability groups of stroke survivors.

## Results

Seventy participants were eligible and agreed to participate in this study. Their baseline characteristics are shown in Table 1. Stroke severity was measured using the National Institutes of Health Stroke Scale (NIHSS). One-way ANOVA showed that the groups did not differ significantly from each other in age, height, weight or BMI (*p* > 0.05).

**Table 1:** Demographic characteristics of the participants.

	Walking ability level				
	Household (*n* = 29)	Limited community (*n* = 21)	Community (*n* = 20)	All (*n* = 70)	*p*
Sex (male: female), *n*	17:12	14:7	12:8	43:27	
Age (yr), mean (SD)	66 (8)	66 (7)	64 (7)	65 (7)	0.5
Height (cm), mean (SD)	165 (9)	166 (8)	165 (8)	166 (8)	0.8
Weight (kg), mean (SD)	64 (10)	67 (10)	69 (10)	66 (10)	0.2
BMI, mean (SD)	24 (3)	24 (3)	25 (4)	24 (3)	0.2
Side of stroke (right: left), n	18:11	6:15	12:8	36:34	
Time since stroke (days), median (range)	57 (10 to 329)	60 (11 to 279)	92 (10 to 311)	71 (319)	
NIHSS (0–42), median (range)	9 (6 to 12)	7 (3 to 10)	2 (1 to 9)	7 (1 to 12)	
10MWT (m/s), mean (SD)	0.2 (0.1)	0.6 (0.1)	1.1 (0.2)	0.6 (0.4)	

BMI, body mass index; NIHSS, National Institutes of Health Stroke Scale; 10MWT, 10-Meter Walking Test.

**Figure 2:**
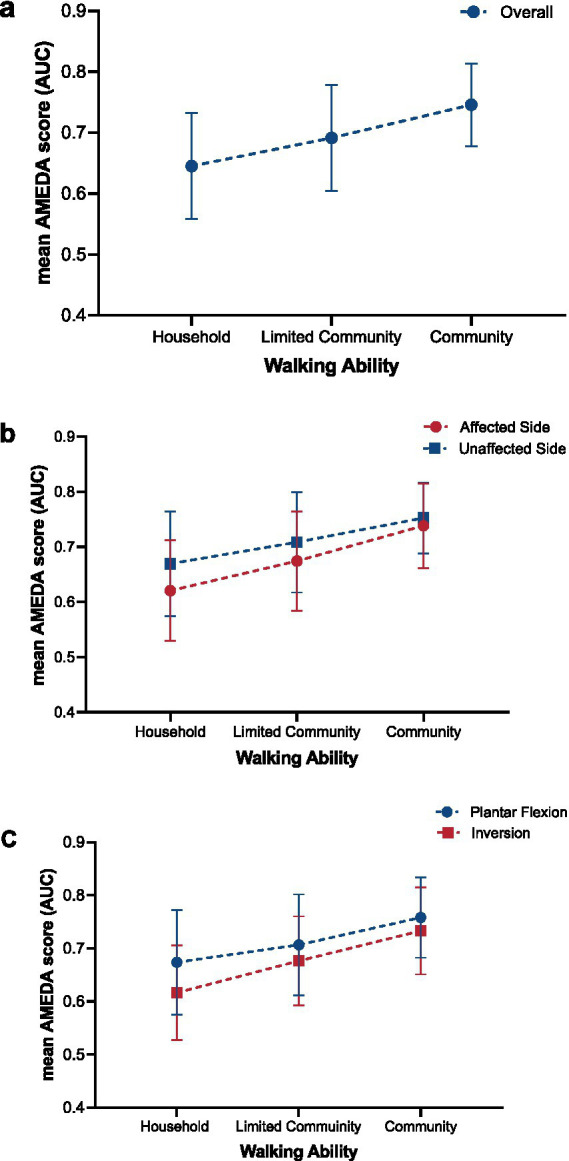
Trends in ankle JPS acuity reflected in AMEDA scores represented as mean AUC values (± SD) for **(a)** overall, **(b)** affected and unaffected side, and **(c)** plantar flexion and inversion across the three different walking ability groups (Household, Limited Community, and Community). Error bars represent standard deviations (SD). JPS, Joint Position Sense; AMEDA, Active Movement Extent Discrimination Assessment; AUC, Area under the Receiver Operating Characteristic (ROC) curve.

The results of the 2x2x3 repeated ANOVA showed that when analyzing the overall mean ankle AMEDA scores, combining both sides and movements, there was a significant linear increase with increasing walking ability among stroke survivors (F_1.67_ = 17.802, *p* < 0.001; Figure 2a). This finding indicates that ankle AMEDA scores increase linearly with walking ability level, highlighting a positive association between walking ability and ankle JPS acuity. There was no significant interaction between Side and Walking ability (F_2.67_ = 1.686, *p* = 0.193, η^2^ = 0.048) or between Movement direction and Walking ability (F_2.67_ = 2.213, *p* = 0.117, η^2^ = 0.062). This lack of interaction indicates that the linear increase in ankle JPS acuity over walking ability level was the same for the affected and unaffected sides, and for plantar flexion and inversion movements.

ANOVA with repeated measures demonstrated a significant difference between the affected and unaffected sides, with ankle JPS acuity for the unaffected side significantly higher than for the affected side (F_1.67_ = 22.508, *p* < 0.001, η^2^ = 0.251; Figure 2b). Post-hoc paired t-tests indicated a significant side difference was found in the Household group and Limited Community group, while there was no significant difference between sides in the Community group (Figure 2b; Table 2).

**Table 2:** Mean AMEDA test AUC scores and standard deviations (SD) for the affected and unaffected sides of the three walking ability groups, with differences between the sides and *t*-test results.

Walking ability	Affected	Unaffected	MD	SEM	t	p
Household	0.62 ± 0.09	0.67 ± 0.09	−0.049	0.012	−3.94	**<0.001**
Limited community	0.67 ± 0.09	0.71 ± 0.09	−0.034	0.011	−3.05	**0.006**
Community	0.74 ± 0.08	0.75 ± 0.06	−0.014	0.010	−1.47	0.158

AMEDA, Active Movement Extent Discrimination Assessment; AUC, Area under the Receiver Operating Characteristic (ROC) curve; MD, Mean Difference; SEM, Standard Error Mean. AUC scores can range from 0.5 (chance) to 1.0 (perfect performance).

Bold values indicate statistically significant results (*p* < 0.05).

The mean ankle AMEDA scores for plantar flexion were significantly higher than for inversion (F_1.67_ = 21.366, *p* < 0.001, η^2^ = 0.242; Figure 2c). Significant differences were also found from post-hoc paired t-tests in the Household group and Limited Community group while no significant difference was found between plantar flexion and inversion scores in the Community group (Figure 2c; Table 3).

**Table 3:** Mean AMEDA test AUC scores and standard deviations (SD) for the plantar flexion and inversion movement directions for the three walking ability groups, with difference between directions and *t*-test results.

Walking ability	Plantar flexion	Inversion	MD	SEM	*t*	*p*
Household	0.67 ± 0.10	0.62 ± 0.09	0.057	0.013	4.44	**<0.001**
Limited community	0.71 ± 0.10	0.68 ± 0.08	0.030	0.010	3.11	**0.006**
Community	0.76 ± 0.08	0.73 ± 0.08	0.025	0.018	1.38	0.185

AMEDA, Active Movement Extent Discrimination Assessment; AUC, Area under the Receiver Operating Characteristic (ROC) curve; MD, Mean Difference; SEM, Standard Error Mean. AUC scores can range from 0.5 (chance) to 1.0 (perfect performance).

Bold values indicate statistically significant results (*p* < 0.05).

A one-way ANOVA was conducted to compare ankle AMEDA scores on both sides of the body between the walking ability groups (household, limited community and community). The results revealed that the walking ability groups differed significantly from each other in ankle JPS acuity (*p* < 0.05), with the only exception being the plantar flexion scores on the unaffected side (*p* = 0.064; Table 4).

**Table 4:** Mean AMEDA test AUC scores and standard deviations (SD) for the affected and unaffected sides tested in the plantar flexion and inversion movement directions with results for ANOVAs between three walking ability groups.

Movements	Sides	Community	Limited community	Household	*F*	*p*
Plantar flexion	Affected	0.75 ± 0.09	0.68 ± 0.10	0.65 ± 0.10	6.15	**0.004**
	Unaffected	0.77 ± 0.08	0.73 ± 0.10	0.70 ± 0.11	2.86	0.064
Inversion	Affected	0.73 ± 0.09	0.67 ± 0.09	0.59 ± 0.10	12.83	**<0.001**
	Unaffected	0.74 ± 0.08	0.69 ± 0.10	0.64 ± 0.10	6.50	**0.003**

AMEDA, Active Movement Extent Discrimination Assessment; AUC, Area under the Receiver Operating Characteristic (ROC) curve; ANOVA, Analysis of Variance. AUC scores can range from 0.5 (chance) to 1.0 (perfect performance).

Bold values indicate statistically significant results (*p* < 0.05).

**Figure 3:**
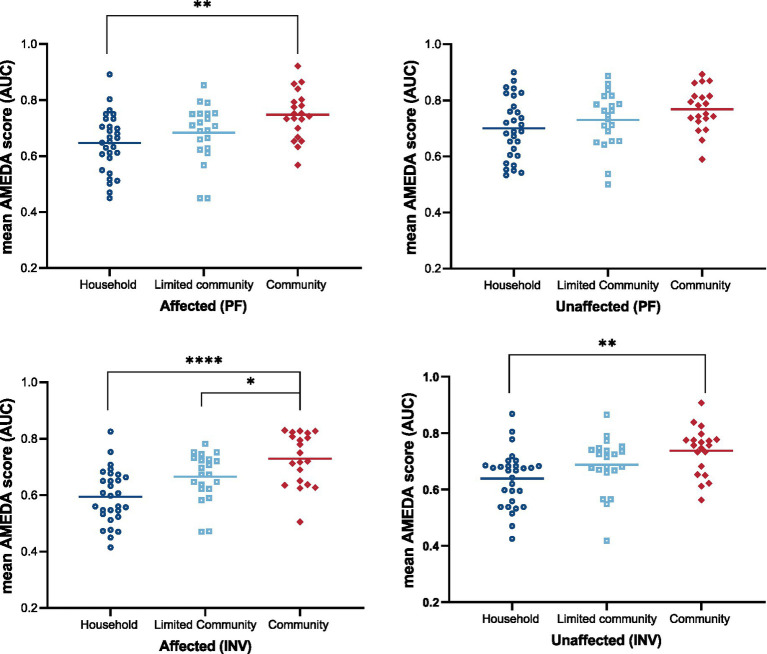
Ankle JPS acuity as mean AMEDA scores (AUC) for groups categorized by walking ability (Household, Limited Community, and Community) on two sides (Affected and Unaffected) for plantar flexion (PF) and inversion (INV). Dots represent individual AMEDA scores, while horizontal lines indicate group means. Statistical significance is indicated as follows: *p < 0.05, **p < 0.01, ****p < 0.0001, all after correction for multiple comparisons. JPS, Joint Position Sense; AMEDA, Active Movement Extent Discrimination Assessment; AUC, Area under the Receiver Operating Characteristic (ROC) curve; PF, plantar flexion; INV, inversion.

Post-hoc tests revealed that there were significant differences in all ankle JPS acuity comparisons between the Household group and Community group, except for unaffected side ankle JPS acuity in plantar flexion, where no difference was seen between these two groups (Figure 3).

## Discussion

The association between ankle JPS acuity, for different sides and movement directions, and walking ability has not been previously determined among stroke survivors, to our knowledge. The current study produced three important findings. Firstly, for people with sub-acute unilateral stroke, ankle JPS acuity on their affected side was consistently lower than that on the unaffected side. Thus, stroke survivors had ankle JPS acuity deficits on the affected side. Further, the affected side ankle JPS acuity deficits were significant among household and limited community stroke survivors, but not in those with unrestricted community mobility levels. Regarding the effect of movement direction on ankle JPS acuity, plantar flexion movements were discriminated better than inversion movements by stroke survivors. Thirdly, stroke survivors with higher walking ability had higher ankle JPS acuity in comparison with household ambulators, regardless of whether the limb tested was the affected or unaffected side, or in the plantar flexion or inversion movement direction.

In this study, household (<0.4 m/s) and limited community (0.4–0.8 m/s) stroke survivors had lower ankle AMEDA scores than community stroke survivors (>0.8 m/s), regardless of side or movement direction. To walk in the community under different conditions, the central nervous system must not only receive sensory information about changes in plantar pressure, joint position and loading but also integrate them to generate appropriate motor responses. Impaired integration of these sensory inputs can disrupt somatosensory processing, leading to decreased gait speed and step length can occur (11–13), which is consistent with our findings. In community ambulators there was no significant difference in ankle JPS acuity between sides or movement directions, whereas household and limited community stroke survivors demonstrated significant differences in each of these factors. One previous study investigated the relationship between stroke survivors with different walking ability and their ability to adjust gait patterns when walking on an artificial grass surface (27). They found similar results, in that only the community ambulators had the ability to adapt their gait on the grass surface by increasing symmetrical step length. Our study may provide evidence for an underlying mechanism for this performance deficit on grass. In comparison with limited and household ambulators, only the community ambulators had achieved the ability to integrate appropriate sensory inputs to accurately guide foot position and achieve symmetry on both sides, which has implications for sensory reception while walking on surfaces such as grass. Another study reported that an uneven surface, such as artificial grass, might provide more somatosensory information from the plantar region than even surfaces (28), a fact which might have contributed to better sensation in community ambulators (29).

After stroke, damage to the somatosensory cortex can result in proprioceptive deficits (30). Disuse might also contribute to negative effects on proprioception after stroke based on the principle of “use it and improve it, or lose it” (31). Household and limited community stroke survivors here demonstrated lower ankle AMEDA scores on the affected side, which indicate plantar tactile (sense of touch) and ankle JPS acuity deficits on the affected sides after stroke. Previous research has identified a strong correlation between plantar tactile sensitivity and ankle JPS acuity (r = 0.8, *p* < 0.001), highlighting the potential influence of tactile impairments on ankle proprioceptive performance (32). Although our study did not directly measure plantar tactile sensitivity, participants performed the AMEDA assessment barefoot, ensuring that plantar tactile inputs were included during testing. A possible explanation of the observed lower AMEDA scores on the affected side may be a use effect involving diminished muscle activation and decreased weight bearing on the affected side (8, 9). Joints on the affected side receive less somatosensory input and motor output in comparison to the unaffected side. As a result, ankle JPS acuity on the affected side is lower than the unaffected side among household and limited community stroke survivors, but not in community ambulators.

Another potential explanation for this finding is that ankle position sense acuity may be influenced by participants’ activity levels. Participants with higher walking abilities, such as community ambulators, are likely to be more functionally active and engage more frequently in activities of daily living (ADL). Increased activity levels could enhance proprioception through both peripheral and central mechanisms (33, 34). At the peripheral level, increased physical activity is known to improve muscle strength, which in turn provides better movement control and improves joint position sense under weight-bearing conditions (33). At the central level, physical activity can modulate mechanoreceptor gain and induce neuroplastic changes in the CNS. To be specific, repetitive afferent inputs from the mechanoreceptors could modify the cortical maps of the body over time. Plastic changes in the cortex can be induced by repeated positioning of body and limb joints in specific spatial position as required by physical activity (34). Over time, regular physical activity can increase cortical representation of the joints, leading to improved position sense acuity. This aligns with the “use it or lose it” principle, suggesting that participants with higher walking abilities, such as community ambulators, may benefit from more frequent proprioceptive engagement.

This study also found that participants with stroke demonstrated higher ankle position sense acuity for movement in the plantar flexion rather than inversion direction, which is consistent with the hypothesis proposed by Refshauge and Fitzpatrick (35) that the sensory input from a given movement is dependent on the number of muscle fibers being stretched. Thus, the first possible reason is that ankle plantar flexion movement involves larger muscles with more muscle fibers, compared with those needed to make an inversion movement. Previously, Lloyd and Caldwell found that practice or use improves proprioception, especially the awareness of movement extent (36). Given that the plantar flexors play an important role in the push-off phase and in the initiation of the swing phase, interventions that practice plantar-flexor function are often included in gait-retraining after stroke (37). Compared with plantar flexion, less attention has been given to ankle inversion. Therefore, the second possible reason for the finding here is that the intensity and duration of sensory interventions focusing on ankle movements were different. Consequently, the AMEDA scores in plantar flexion were higher than they were in inversion.

The ability to detect differences in inversion movement extent may have real-world significance. Errors in extent of inversion movement are associated with adjusting the degree of ankle inversion used during the trip-vulnerable and mid-stance swing phase (38). In addition, impaired ankle inversion JPS acuity is associated with increased risk of falls and fear of falling during walking in older adults (39). Therefore, more practice programs designed to improve ankle JPS acuity would be appropriate interventions to refine use of sensory inputs after stroke.

Currently, systematic reviews suggest that proprioceptive training involving both active and passive movements, with or without visual feedback, is effective as an intervention to improve sensorimotor function in healthy adults (40). However, there are few studies about lower limb proprioception training for stroke survivors. It seems possible that, after participating in specific sensory training, individuals with stroke could more successfully improve their motor performance (15, 41, 42). Park et al. (41) demonstrated a significant increase in balance and gait ability after a six-week ankle proprioceptive control program among chronic stroke participants. In this study, TUG scores showed a significant speed improvement from 20.47 s (0 week), 17.9 s (4 week) to 15.27 s (6 week) and step length showed statistically significant increases on the affected side from 38.88 cm (0 week) to 44.12 cm (4 week) and 45.16 s (6 week). These results suggest that an ankle proprioceptive control program focusing on somatosensory sensation could be effective if employed to improve stroke survivors’ balance and walking abilities. However, more research studies are needed to explore the effectiveness of post-stroke ankle proprioception training. The findings of our study highlight the important role of ankle proprioception (JPS acuity) in walking after stroke, but whether walking can be regarded as an exercise to improve foot and ankle JPS acuity needs further exploration.

This study has some limitations. First, the sample size was modest, even though it satisfied the sample size calculation and significant findings were observed. More participants may increase the generalizability of the results. Further, participants in this study were mild to moderate stroke survivors and able to walk at least 10 m. We did not include those stroke survivors who were unable to bear weight and walk, and future study should investigate ankle JPS acuity in these populations.

Third, the AMEDA test used in this study specifically measures ankle JPS acuity. However, other domains of proprioception, such as position sensitivity, motion sense, and force sense were not assessed. These additional components of proprioception are important for a comprehensive understanding of sensorimotor function and may significantly influence walking ability. Therefore, future studies should consider incorporating assessments that evaluate these broader aspects of proprioception (43), such as the movement reproduction test to evaluate movement discrimination sense and the force reproduction test to assess force discrimination sense, to better understand how proprioceptive deficits affect motor performance after stroke. Additionally, the AMEDA evaluates ankle JPS acuity in a weight-bearing and standing position. Developing and measuring ankle JPS acuity under dynamic conditions, such as during walking, to better capture the functional relevance of ankle JPS acuity deficits in stroke survivors should be considered in future research.

Finally, the primary analysis of this study did not include the effects of stroke severity (using NIHSS), stroke type or lesion location on changes in post-stroke ankle JPS acuity. Studies with larger sample sizes are warranted to address this important consideration and to explore these relationships more comprehensively. Furthermore, although this study did not investigate individual-level correlations between ankle JPS acuity and gait speed, such analyses could provide valuable insights and should be considered in future research.

### Implications for physiotherapy practice

Overall, this study supports the need for addressing ankle JPS acuity deficits in post-stroke rehabilitation implemented to improve walking ability and functional recovery. The finding that stroke survivors demonstrated higher ankle JPS acuity in plantar flexion compared to inversion movements highlights the need for movement-specific assessments and interventions.

Compared to traditional assessments, such as the Rivermead Assessment of Somatosensory Performance (RASP) or joint position reproduction (JPR) tests, which are conducted in non-weight-bearing positions, the AMEDA evaluates ankle JPS acuity in a weight-bearing and standing position. This approach enhances the ecological validity of the test and better reflects the functional demands in daily activities.

The findings of this study also support the practicality and safety of using the AMEDA to assess and monitor ankle JPS acuity after stroke. The observed bilateral deficits in ankle JPS acuity, particularly in those with lower walking ability (household group), indicate the importance of assessing both the affected and unaffected sides. Measuring both affected and unaffected sides enables clinicians to monitor changes in ankle JPS acuity over time and assess the effectiveness of tailored clinical interventions.

## Conclusion

Ankle JPS acuity on the affected side was lower than the unaffected sides among individuals with unilateral hemisphere stroke. Compared with inversion movements, stroke survivors had higher ankle JPS acuity in making plantar flexion movements. Overall, stroke survivors with better ankle JPS acuity tended to have higher walking ability, highlighting the importance of ankle JPS acuity in walking ability after stroke. These findings provide new insights into proprioceptive deficits after stroke and their relevance in neurorehabilitation.

## Data Availability

The original contributions presented in the study are included in the article/supplementary material, further inquiries can be directed to the corresponding authors.
